# Characterization of a Functional Hydrogel Layer on a Silicon-Based Grating Waveguide for a Biochemical Sensor

**DOI:** 10.3390/s16060914

**Published:** 2016-06-18

**Authors:** Yoo-Seung Hong, Jongseong Kim, Hyuk-Kee Sung

**Affiliations:** 1 School of Electronic and Electric Engineering, Hongik University, Seoul 121-791, Korea; yoosing87@gmail.com; 2 Department of Neurology, Molecular Imaging and Neurovascular Research (MINER) Laboratory, Dongguk University Ilsan Hospital, Goyang 10326, Korea

**Keywords:** waveguides, diffraction gratings, biological sensing and sensors, optical sensing and sensors

## Abstract

We numerically demonstrated the characteristics of a functional hydrogel layer on a silicon-based grating waveguide for a simple, cost-effective refractive index (RI) biochemical sensor. The RI of the functional hydrogel layer changes when a specific biochemical interaction occurs between the hydrogel-linked receptors and injected ligand molecules. The transmission spectral profile of the grating waveguide shifts depends on the amount of RI change caused by the functional layer. Our characterization includes the effective RI change caused by the thickness, functional volume ratio, and functional strength of the hydrogel layer. The results confirm the feasibility of, and set design rules for, hydrogel-assisted silicon-based grating waveguides.

## 1. Introduction

The quantification of a refractive index (RI) change has been shown to be an effective method of developing a biochemical sensor that measures biomolecular interactions [1,2,3,4]. The method exhibits significant advantages such as no fluorescent labeling, high throughput, and improved sensitivity than other biochemical sensors [5,6,7,8,9]. Several approaches have been proposed and successfully demonstrated to quantify the RI change induced by the concentration change of biochemical molecules. They include surface plasmon resonance (SPR) [10], ring resonators [11], long-period fiber grating [12], a grating coupler [13], grating waveguides [14], and metallic photonic crystal sensors [15,16]. The proposed techniques utilize the resonance frequency shift of a transmission spectral profile that occurs when a fraction of the guided mode interacts with receptors and targeted molecules on the waveguide surface [4,5,17]. Among these techniques, a silicon-based grating waveguide is especially promising because of its fabrication compatibility with the standard complementary metal-oxide-semiconductor (CMOS) processes, the simplicity of the structure, and the corresponding cost competitiveness as well as its superior sensitivity [14]. Pham *et al.* utilized the shift of a sharp fringe in the transmission spectrum near the stop-band edge of the grating [14]. They successfully demonstrated a direct and label-free protein biosensor based on the grated waveguide.

Detection and quantification of the multivalent binding of proteins is a crucial step for a better understanding of the fundamental mechanisms in the immune system, cancer, and thrombosis [18,19,20]. To detect multivalent binding efficiently as well as to distinguish it from the monovalent case, Kim *et al.* utilized hydrogel micro-particles as a functional layer [21]. They successfully demonstrated the RI change of the microgels through a ligand-induced receptor dimerization that accompanies the multivalent binding process. Typically, the capability of distinguishing multivalent binding from monovalent is limited in most current analytical technologies without labeling fluorescence molecules. Isothermal titration calorimetry (ITC) could be used, but this consumes a relatively large amount of purified proteins. On the other hand, when microgels containing appropriate receptors are used as functional layers, their RI changes significantly because of a dimerization between injected target proteins and receptors, and a corresponding local deswelling of the functional layer [21]. It has been reported that the RI of the functional microgel layer changes by 0.05 from its original value because of the multivalent binding and the deswelling of the microgel layer [22].

Herein, we propose a compact and inexpensive biochemical sensor prototype using a silicon-based grating waveguide assisted by a hydrogel top cladding layer as a functional layer. We perform a numerical simulation focusing on the characterization of the functional layer that is dedicated to detecting and quantifying the multivalent binding of proteins. The proposed biochemical sensor consists of a typical silicon-on-insulator (SOI) bottom layer, a silicon-based grating waveguide core, and a hydrogel functional layer on top of the grating waveguide. The waveguide core is designed as a grating structure to achieve Fabry–Perot resonances of the Bloch modes. The transmission spectral profile exhibits multiple resonance peaks due to optical interference between guided modes in the grating waveguide. The RI of the hydrogel layer changes as a result of a biochemical interaction (e.g., multivalent binding of proteins) between the functional layer, receptor, and targeted molecules. Consequently, the effective RI of the waveguide changes and is followed by a shift in the resonance wavelength of the spectral transmission profile. We calculated the waveguide effective RI as a function of several parameters of the functional layer. These parameters include the thickness, functional volume ratio, and functional strength of the hydrogel layer. We determined the optimized hydrogel layer thickness when a double-layer model was employed. We calculated the dynamic range of the waveguide effective RI and performed a comparison between single- and double-layer models. The results demonstrate the feasibility of, and set design rules for, silicon-based grating waveguide sensors assisted by a functional hydrogel layer. The proposed configuration is a promising candidate for a low-cost, CMOS-compatible sensor for detecting the multivalent binding of proteins.

## 2. Principles

### 2.1. Silicon-Based Grating Waveguide Sensor

**Figure 1:**
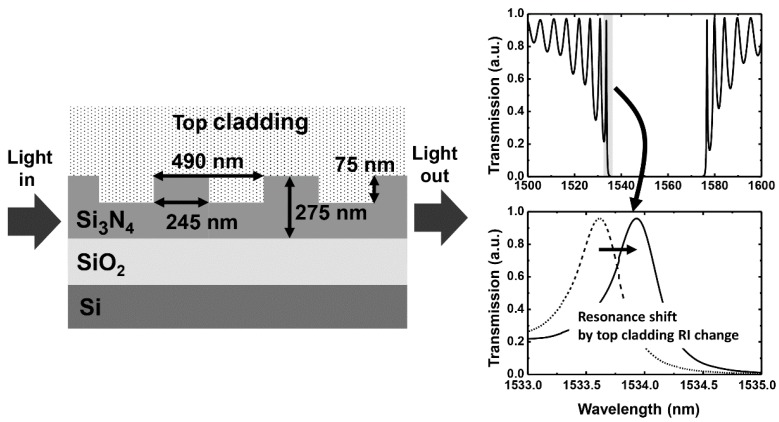
Schematic of a grating waveguide for a refractive index (RI) sensor and its sensing principle.

Figure 1 shows the schematic of a grating waveguide structure and its sensing principle. The waveguide core consists of Si_3_N_4_ on top of a SiO_2_ bottom cladding layer. A 200-period structure, having a 490-nm pitch with 50% duty cycle and 75-nm etch depth, is used as a grating waveguide. The transmission profile near the 1550-nm wavelength is shown in the inset of Figure 1. Fabry–Perot resonance modes with a stopband are formed by the distributed feedback nature of the periodic grating structures. The resonance mode closest to the stopband exhibits the narrowest bandwidth (*i.e.*, the highest quality factor), so that a high sensitivity can be achieved by utilizing the fringe mode. When the RI of the top cladding changes, the effective RI of the core waveguide changes, causing the transmission profile to shift [23]. We carried out a numerical investigation of the grating waveguide using a 2-D finite-difference time-domain (FDTD) method and a finite-difference method (FDM) for transverse electric (TE) mode using software from Lumerical Solutions, Inc. (Vancouver, BC, Canada). We incorporated a 2-D FDTD simulation-window of 110 µm in the x-direction (propagation direction) and 4 µm in the y-direction (cross-section direction), using perfectly matched layers in boundary conditions for a grating waveguide of 98 µm by 275 nm. In order to obtain accurate transmission of the grating waveguide, a mesh grid size of ∆x = 40 nm in the x-direction and ∆y = 4 nm in the y-direction as well as a time-step size of ∆t = 1.3 × 10^−17^ s were used in the FDTD simulation.

**Figure 2:**
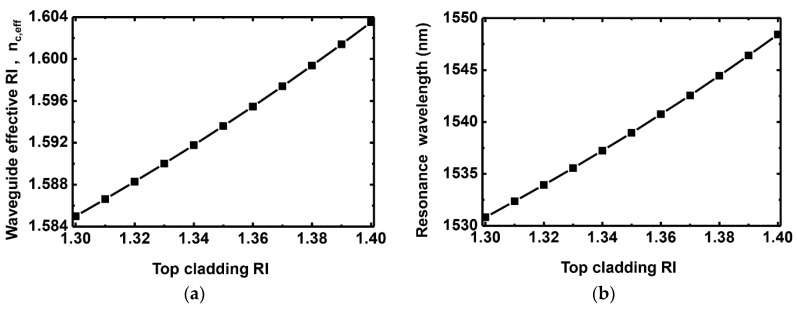
(**a**) Effective RI of the waveguide core and (**b**) resonance wavelength of a fringe mode as a function of the top cladding RI.

Figure 2a shows the simulation result for the effective RI, $n_{c,eff}$, of a waveguide as a function of the top cladding RI for the structure shown in Figure 1. Here, $n_{c,eff}$ is defined as a spatial average of the effective RIs of the entire waveguide core [24]. When the top cladding RI varies, $n_{c,eff}$ changes as shown in Figure 2a. Figure 2b shows the corresponding resonance wavelength of the fringe mode. The sensitivity of the grating waveguide is 176 nm/RIU, where RIU stands for a refractive index unit. The result coincides with the relationship between $n_{c,eff}$ and resonance wavelength given by [25]. In the Bragg grating structure, the resonance wavelength $\lambda_{B}$ is written as [25]

(1) $$\lambda_{B}=2\Lambda n_{c,eff}$$

where Λ is a grating period. The corresponding shift of the Bragg resonance wavelength $\lambda_{B}$ is given by

(2) $$\Delta \lambda_{B}=2\Lambda \Delta n_{c,eff}$$

The resonance shift is attributed to evanescent waveguide modes that penetrate and interact with the top cladding layer. As shown in Figure 2a,b, the resonance shift of the transmission response of the grating waveguide is directly related to the change in the waveguide effective RI. Therefore, we calculate the waveguide effective RI using FDM instead of the resonance shift in this paper to provide physical insight as well to save calculation effort. The figure-of-merit (FOM) of the RI sensor can be calculated by dividing the sensitivity by the full-width at half-maximum (FWHM) of a Fabry–Perot resonance mode. Although the sensitivity is not great compared with some previous work [16], the narrow transmission resonance of the Fabry–Perot mode provides a high FOM of 400. It should also be noted that, although the calculated sensitivity in Figure 2b might be slightly lower than that of other RI sensors in [4], the significant top cladding RI change of a functional hydrogel layer can provide a sufficient change of the waveguide effective RI [22,26], and the silicon-based grating waveguide combined with a functional layer can be a promising solution to enable a low-cost, compact, CMOS-compatible biochemical sensor platform.

### 2.2. Modeling of a Functional Layer for Sensing the Multivalent Binding of Proteins

**Figure 3:**
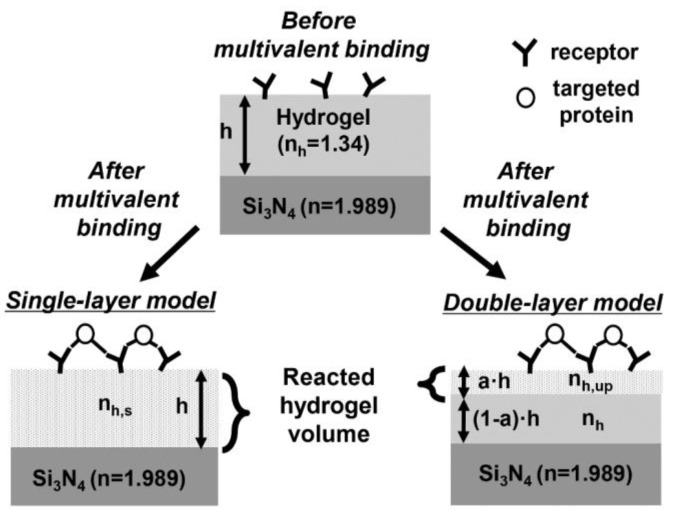
Conceptual diagram showing the RI change of a functional hydrogel layer after the multivalent binding of proteins. A single-layer and a double-layer model are considered for comparison. h: hydrogel layer thickness; a: functional volume ratio, $n_{h}$: RI of a hydrogel layer before the multivalent binding or RI of the non-reacted hydrogel volume after the multivalent binding; $n_{h,up}$: RI of the reacted hydrogel volume after the multivalent binding.

Figure 3 shows the conceptual diagrams of the grating waveguide with a functional hydrogel layer for sensing multivalent binding of proteins. A Si_3_N_4_ grating waveguide with the same dimensions as that of Figure 1 is used as a waveguide core, and a hydrogel layer on top of the waveguide serves as the functional layer. When the receptors in the hydrogel layer are exposed to ligand proteins, ligand-induced receptor dimerization occurs, yielding a local deswelling of the hydrogel layer. Subsequently, the RI of the hydrogel layer is reported to change by about 0.05 [22]. The local deswelling of the hydrogel layer typically takes place at its top surface. Therefore, both single- and double-layer models are considered to provide accurate characterization and design guidelines for the functional layer. In the single-layer model, the RI of the functional hydrogel layer $n_{h}$ is assumed to change uniformly to $n_{h,up}$ as a result of the multivalent binding of proteins. In contrast, the RI of the hydrogel layer in the double-layer model is assumed to form two separate values, $n_{h,up}$ and $n_{h}$, respectively, where $n_{h,up}$ is the RI of the reacted hydrogel that results from the multivalent binding process. Only the upper layer RI changes while the bottom layer RI remains the same as its original value $n_{h}$. A functional volume ratio a is defined as the ratio of the reacted volume to the total hydrogel volume. Note that the effect of biological crosslinking on hydrogel shrinkage is significantly lower than that of hydrogel volume phase transitions caused by changes in temperature, pH, and ionic strength [27,28].

## 3. Simulation Results

**Figure 4:**
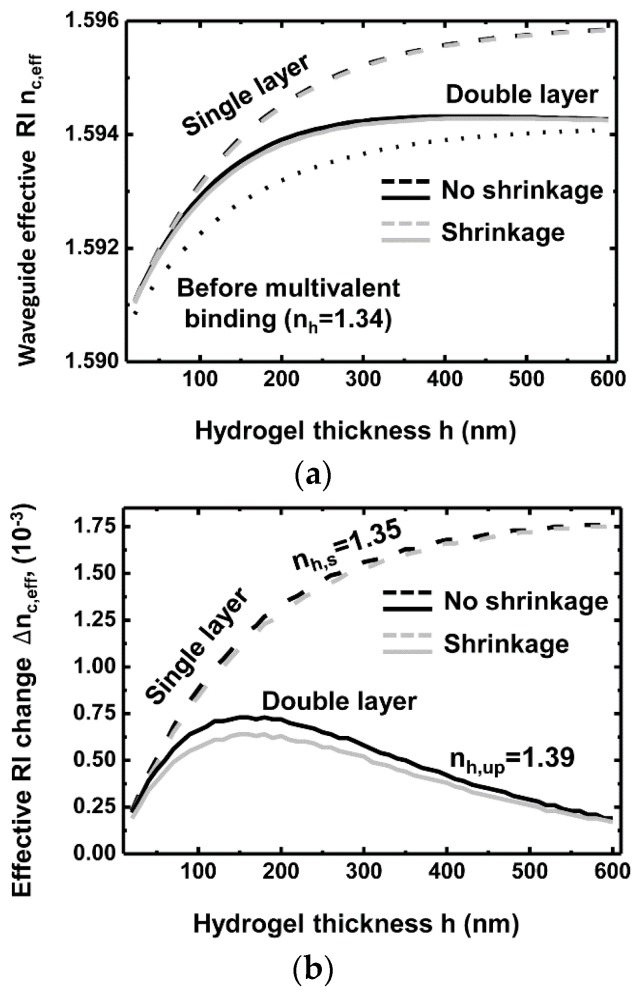

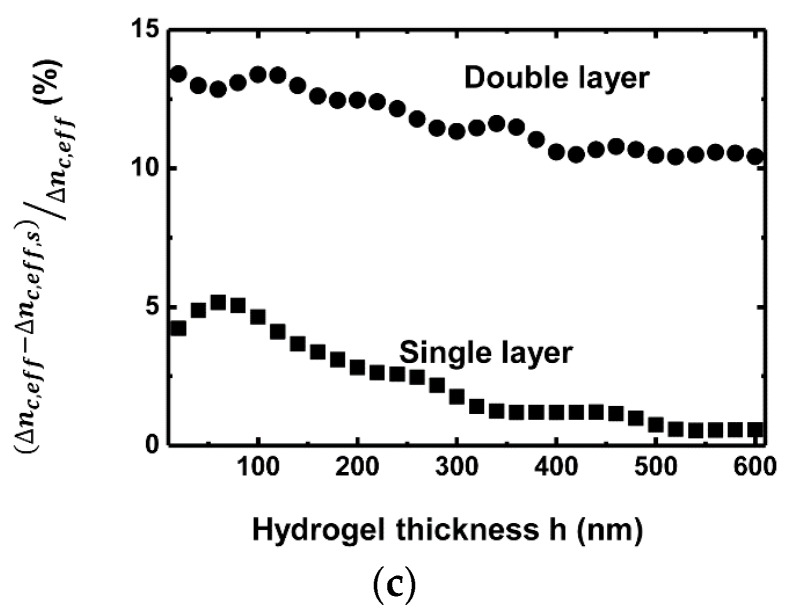
(**a**) Waveguide effective RI $n_{c,eff}$ for various hydrogel thicknesses (dotted curve: before functionalization, solid curve: after functionalization using a double-layer model ($n_{h,up}$ = 1.39, $n_{h}$ = 1.34), dashed curve: after functionalization using a single-layer model ($n_{h,s}$ = 1.35)) and (**b**) effective RI change $n_{c,eff}$ for various hydrogel thicknesses (solid curve: double-layer model ($n_{h,up}$ = 1.39, $n_{h}$ = 1.34), dashed curve: single-layer model ($n_{h,up}$ = 1.35)). The functional volume ratio a is 0.2 for the double-layer model. The black and gray curves represent the cases with and without considering hydrogel shrinkage after deswelling. Shrinkages of 2% and 10% in thickness are assumed for a single- and double-layer model, respectively. (**c**) Effect of the hydrogel shrinkage on the waveguide effective RI change for single- and double-layer models.

Figure 4a shows the waveguide effective RI $n_{c,eff}$ as a function of the thickness of the hydrogel layer for single- and double-layer models. The RI of the Si_3_N_4_ waveguide and SiO_2_ bottom cladding layers are 1.989 and 1.444, respectively. All the RIs and effective RIs in this paper are specified at a wavelength of 1550 nm. The RI of the hydrogel layer before the multivalent binding $n_{h}$ is 1.34. It is assumed to change to 1.35 for the single-layer model (=$n_{h,s}$) after the multivalent binding. In the double-layer model, the RI of the reacted hydrogel $n_{h,up}$ changes to 1.39, while that of the non-reacted layer remains at 1.34 as previously reported on the basis of experimental observations [21,26,29]. The functional volume ratio a is set at 0.2 for the double-layer model, so that the volume RI change remains the same for both the single- and double-layer models. Before the multivalent binding of proteins, $n_{c,eff}$ increases with the hydrogel thickness owing to the extension of the region where evanescent fields exist (dotted curve). After the multivalent binding, $n_{c,eff}$ also increases with the hydrogel thickness for both the single-layer (black dashed curve) and double-layer (black solid curve) models. In the double-layer model, the increase of $n_{c,eff}$ starts to saturate at about 300 nm because surface sensing utilizing evanescent fields becomes less effective as a result of the increased non-reacted volume. The $n_{c,eff}$ of the single-layer model is larger than that of the double-layer model because the entire volume is reacting in the single-layer model. Therefore, the bulk hydrogel with a uniform RI change in the entire volume is preferable to achieve a higher sensitivity. We also consider the shrinkage effects of hydrogel (gray dashed and gray solid curves, respectively) after multivalent binding on the basis of previous studies [27]. We assume that the maximum thickness change of the reacted hydrogel layer is 10% based on the previous report. For a single-layer model, we assume a 2% decrease in a hydrogel thickness because an equal amount of biological reaction is assumed for double-layer (20% of reactive volume) and single-layer models (100% of reactive volume). This results in the maximum thickness changes of the reacted hydrogel layer being 2% and 10% for the single- and double-layer model, respectively. Note that the functional volume ratio of the double layer model is 0.2. The solid- and dashed-gray lines show $n_{c,eff}$ considering the shrinkage, while the black lines exhibit $n_{c,eff}$ without considering the shrinkage for single- or double-layer models, respectively. The two gray lines present trends similar to the corresponding black lines while exhibiting a slightly lower value because of the thickness decrease after the deswelling.

Figure 4b shows the corresponding waveguide effective RI difference, $\Delta n_{c,eff}$, before and after the multivalent binding for the shrinkage and non-shrinkage cases. The $\Delta n_{c,eff}$ of the single-layer model is larger than that of the double-layer model, and the $\Delta n_{c,eff}$ of the double-layer model reaches its maximum at a thickness of about 150 nm. This result determines the optimal thickness of the functional layer that is needed to ensure that the multivalent binding produces the highest possible change in effective RI $\Delta n_{c,eff}$. In considering the shrinkage effects of hydrogel due to multivalent binding, we observed almost the same results for the single-layer model and slightly lower effective RI changes for the double-layer model than those of non-shrinkage cases. Hydrogels undergo a volume phase transition upon changes in temperature, pH, and ionic strength, which induces dramatic changes in volume and optical density. Thus, one can easily measure the changes using Dynamic Light Scattering (DLS) or optical microscopy. In contrast, biological crosslinking causes significantly smaller changes in volume and thus is hardly detectable using DLS. We have calculated the effects of hydrogel shrinkage on waveguide effective RI with and without shrinkage owing to multivalent binding as shown in Figure 4c. It shows the effect of the shrinkage on top of the RI change by the deswelling. The effect of the shrinkage is defined as ($\Delta n_{c,eff}-\Delta n_{c,eff,s}$)/$\Delta n_{c,eff}$, where $\Delta n_{c,eff}$ is the difference of waveguide RI before and after deswelling without considering hydrogel shrinkage, and $\Delta n_{c,eff,s}$ is the difference of waveguide RI before and after deswelling considering hydrogel shrinkage. The effect of the shrinkage ranges from about 2% to 5% and 11% to 13% depending on an initial hydrogel thickness for a single-layer and double-layer model, respectively. The results suggest that the thinner hydrogel layers experience more shrinkage effects. In the double-layer model, the shrinkage effects are much more significant than in the single-layer case because the thickness changes more in the double-layer model.

**Figure 5:**
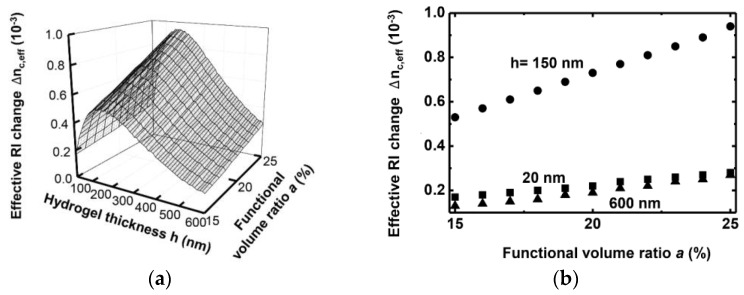
(**a**) $\Delta n_{c,eff}$ as a function of the functional volume ratio a for various hydrogel thicknesses and (**b**) $\Delta n_{c,eff}$ for specific hydrogel thicknesses of 20, 150, and 600 nm. The RI of the reacted hydrogel $n_{h,up}$ has a constant value of 1.39 for all a.

In actual biochemical applications, the functional volume ratio a can be controlled by changing the layer composition properties, applying various receptors, or both. Figure 5a shows $\Delta n_{c,eff}$ for various hydrogel thickness and functional volume ratios. In all further calculations in this paper, hydrogel shrinkage is not considered because the shrinkage does not seem to significantly affect the major trend of the waveguide effective RI change. The value of $\Delta n_{c,eff}$ reaches its maximum at a thickness of about 150 nm, coinciding with the result shown in Figure 4a. It increases as a becomes larger, as shown in Figure 5b. The value of $n_{h,up}$ is assumed to be a constant of 1.39 for all a. The largest $\Delta n_{c,eff}$ was exhibited by the 150-nm-thick layer, and it increased monotonically for a values from 15% to 25% as shown in Figure 5b.

**Figure 6:**
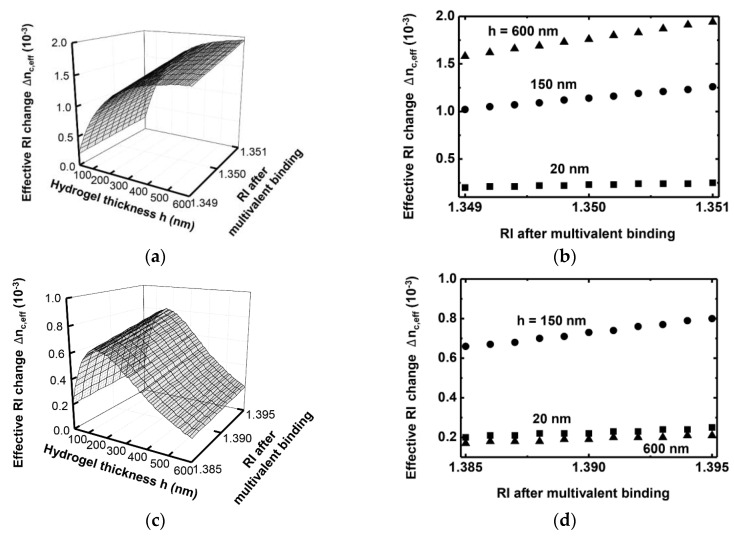
$\Delta n_{c,eff}$ within a small RI variation of the functional layer for various hydrogel thicknesses for the (**a**) single-layer and (**c**) double-layer models. $\Delta n_{c,eff}$ for specific hydrogel thicknesses of 20, 150, and 600 nm for the (**b**) single-layer and (**d**) double-layer models.

We performed a more precise quantification on the $\Delta n_{c,eff}$ within a small RI variation of the hydrogel. Here, the RI of the hydrogel layer varies from 1.385 to 1.395 for both the single- and double-layer models as shown in Figure 6a–d. For the single-layer model, a thicker functional layer provides a larger $\Delta n_{c,eff}$ that yields a larger dynamic range of $\Delta n_{c,eff}$. For the double-layer model, a 150-nm thickness provides the largest $\Delta n_{c,eff}$, which corresponds to the results shown in Figure 4a and Figure 5a. The amount and variation of $\Delta n_{c,eff}$ of the double-layer model is smaller than that of the single-layer model because the grating waveguide is essentially a surface sensor that detects a biochemical process occurring at the waveguide top surface. Therefore, a larger dynamic range of $\Delta n_{c,eff}$ is attained by the single-layer than by the double-layer model. It will be an interesting future study to characterize and compare quantitative signals between monovalent and multivalent bindings. Each binding may produce a significantly different amount of effective RI change in a waveguide.

## 4. Conclusions

We evaluated the characteristics of a functional hydrogel layer on a silicon grating waveguide for use as a simple, low-cost, CMOS-compatible biochemical sensor. Such functionalization of the hydrogel layer provides a significant RI change in response to a specialized biochemical interaction, namely, the multivalent binding of proteins. Characterization of the functional layer was performed numerically by exploring effective RI changes of the proposed grating waveguide including both a single- and double-layer model of the hydrogel. The effective RI change of the waveguide is larger for the single-layer model than for the double-layer model because the surface sensing mechanism utilizes a waveguide evanescent field. We found that an optimal thickness exists for the double-layer model. Our results demonstrate that the waveguide effective RI difference between before and after the multivalent binding process increases with the functional volume ratio. The investigation of a waveguide effective RI caused by a small RI variation of the functional layer allows us to obtain the dynamic range of the proposed biochemical sensor. The characterization of the functional hydrogel layer could be the foundation for the application of a silicon-based grating waveguide sensor to a wide variety of biochemical sensors.
